# Methodology for the remote transfer of GPS receiver station data through a GSM network

**DOI:** 10.1016/j.heliyon.2021.e08330

**Published:** 2021-11-05

**Authors:** Salsabeel E. Othman, Gerges M. Salama, Hesham.F.A. Hamed

**Affiliations:** a Coastal Research Institute, 15 El Pharaana Street, El Shallalat, Alexandria, 21514, Egypt; b Communications and Electronics Department, Faculty of Engineering, Minia University, Minia 61111, Egypt; c Communications and Electronics Department, Egyptian Russian University, Egypt

**Keywords:** APN function, GPS measurements, GPS devices, GPS observations, GPS receiver, Remote access

## Abstract

Coastline alterations severely impact the socio-economic conditions of populations living in coastal regions. Climate changes, together with land subsidence considerations, have increased recently according to high-accuracy fixed tide gauges and land subsidence sensors. In addition, networks of measurement devices are spread throughout the oceans, seas, and coastal areas to capture ongoing changes and to predict future impacts. However, some of these devices still require the in-situ extraction of data for postprocessing. This increases the cost, wastes time, and increases the probability of human errors leading to inaccurate results. This study presents a developed approach to remotely access the Trimble NetR9 GPS receiver device that is fixed at the Coastal Research Institute station in the city of Rosetta in Egypt. This will ease the remote retrieval of the station data for its processing and interpretation.

## Graphical abstract

**Figure:**
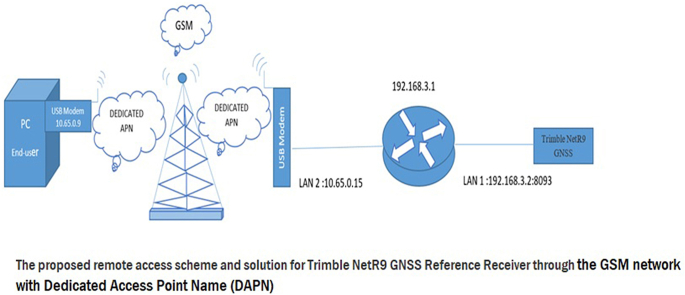

## 1 Introduction

Coastline changes occur as the result of natural and anthropogenic processes and activities [1]. Natural conditions include waves, wind, currents, erosion, and accretion, while human activities involve changes in land use, regulatory structures over rivers, and coastal protection structures [2]. Natural conditions are more prominent with respect to coastline changes because they play an essential role in reshaping the coastline [3, 4].

The monitoring of sea-level variations, tides, and currents, in addition to the vertical land displacement, is an extremely important field of study for numerous applications, such as port planning and operations, government defense, and integrated coastal zone management projects [5]. Coastal ecological studies and predicting sea conditions are increasingly critical to those who reside in coastal areas to help preserve lives and material possessions. In addition, as stated by Tao et al., water level predictions are crucial for maritime administration and the protection of water transport [6].

The regular monitoring of coastal areas, consideration of their morph-dynamics, and identification of the processes influencing sediment transport are essential to better understand the changes and evolutionary trends in coastal systems. This requires a multidisciplinary attitude involving researchers with knowledge in coastal processes and state-of-the-art observation technologies, in addition to reliable data, taking into consideration cost and time limitations [6].

**Figure 1:**
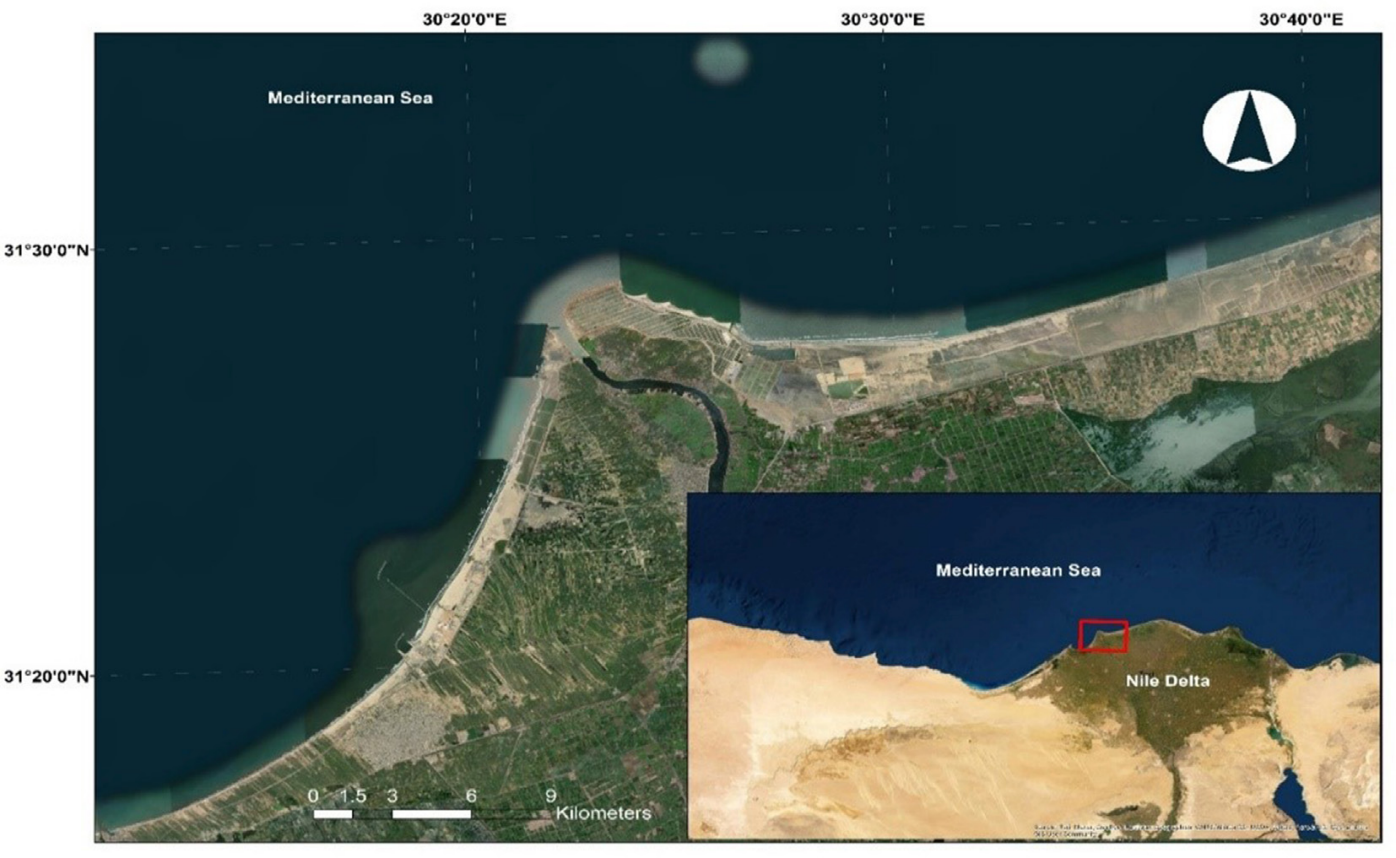
Location of Rosetta.

Rosetta (Figure 1) is a coastal city in Egypt. Its importance lies in its large population, recreation and tourism sites, fisheries, and wide agricultural lands, which are crucial to the Egyptian economy. Sea-level rise and land subsidence are among the threats contributing to the alterations in the coastline of Rosetta. Accordingly, the Coastal Research Institute (CoRI) regularly monitors these changes along with other parameters. CoRI uses GPS technology to measure the land subsidence. According to Wang et al. high-accuracy GPS technology has been used for monitoring land subsidence since the late 1980s [7]. In addition, CoRI uses a set of tide gauges to measure the sea-level rise to accurately calibrate the rates of vertical land movement, as mentioned by Wöppelmann et al. who highlighted the significance of computing the ratio of each process that contributes to the vertical land displacement and subsequently to the projected sea-level variations [8].

### 1.1 GPS receiver position mathematics

GNSS systems use advanced orbital satellite and radio signal technologies to send navigation receivers, via radio signal indications, the time of transmission and the identity of the transmitting satellite. To compute the position of the receiver from these signals, several mathematical operations are required.

**Figure 2:**
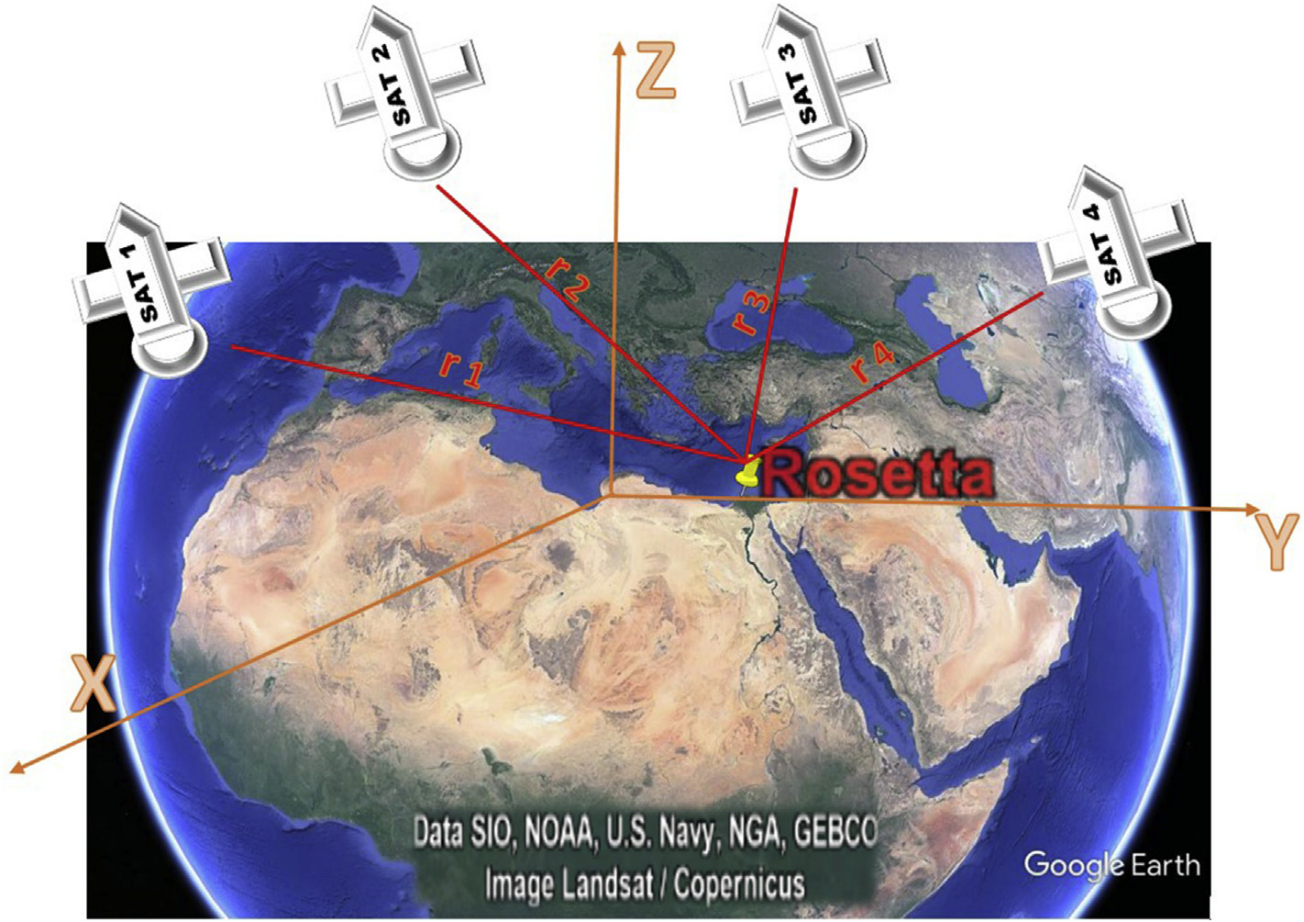
GPS receiver position related to four different satellites.

To estimate the GPS receiver position, it is necessary to receive time signals from four separate satellites and to measure the signal travel times [9]. Therefore, a geocentric three-dimensional coordinate system is used. This three-dimensional rectangular coordinate system has its origin at the center of mass of the Earth, as shown in Figure 2 [10].

The distance r from the satellite to the Rosetta GPS receiver station is [9]

(1) $$r=\sqrt{{\left(X_{\mathrm{SAT}\text{\_i}}-X_{\text{GPS}}\right)}^{2}+{\left(Y_{\text{SAT\_i}}-Y_{\text{GPS}}\right)}^{2}+{\left(Z_{\mathrm{SAT}\text{\_i}}-Z_{\text{GPS}}\right)}^{2}}$$

An incorrect distance is measured, which is known as the pseudo distance or pseudo range (PSR [9]

(2) $$\mathrm{PSR}=r+\Delta t_{0}.C$$

where c is the speed of light and Δt_0_ is the difference between the satellite clock and the receiver clock.

Using Eqs. (2) and (1), the four unknown variables (**Δt**_**0**_, **X**_GPS_, **Y**_GPS_, and **Z**_GPS_) for the four satellites (i = 1 … 4) are determined as follows: [9]

(3) $$\mathrm{PS}R_{i}=\sqrt{{\left(X_{\mathrm{SAT}\text{\_i}}-X_{\text{GPS}}\right)}^{2}+{\left(Y_{\mathrm{SAT}\text{\_i}}-Y_{\text{GPS}}\right)}^{2}+{\left(Z_{\mathrm{SAT}\text{\_i}}-Z_{\text{GPS}}\right)}^{2}}+\Delta t_{0}.C$$

Because of the nonlinearity of the equations, Scarfe et al. computed the root function (which was first linearized according to the Taylor model) and estimated the position of the GPS receiver (**X**_**GPS**_, **Y**_**GPS**_, **Z**_**GPS**_) [9].

Even though the positions of the points in a satellite survey are computed in the geocentric coordinate system, these positions are inconvenient for use by geomatics engineers. Ghilani and Wolf have illustrated three reasons for not using these positions. The geocentric coordinates (X, Y, Z) are accordingly changed to geodetic coordinates of latitude (φ), longitude (λ), and height (h). The geodetic coordinate system (φ, λ, h) [10] is

(4) $$X_{P}=\left(R_{N}+h_{p}\right)\cos\varphi_{P}\cos{\text{λ}}_{P}$$

(5) $$Y_{P}=\left(R_{N}+h_{p}\right)\cos\varphi_{P}\sin{\text{λ}}_{P}$$

(6) $$Z_{P}=\left[R_{N}\left(1-e^{2}\right)+h_{\text{P}}\right]\sin{\text{λ}}_{P}$$

where R_N_ is the radius in the prime vertical of the ellipsoid at point P with a value of 6,387,440.3113 m, *e* indicates the eccentricity of the WGS84 reference ellipsoid with a value of 0.08181919084, and *a* is the secondary axis with a value of 6,378,137 m in the WGS84 reference ellipsoid [10, 11].

(7) $$R_{N}=\frac{a}{\sqrt{1-e^{2}{\sin\;}^{2}\varphi_{P}}}$$

(8) $$\varphi_{P}=\tan^{-1}\left[\frac{Z_{P}+e^{2}R_{N}\sin\varphi_{0}}{{\text{D}}_{P}}\right]$$

Here, φ_**0**_ is the approximate latitude.

This study develops a new approach for the remote access of the land subsidence measuring devices installed in Rosetta. This approach will facilitate the immediate remote retrieval of the recorded data eliminating the need for field visits. This in turn will reduce the costs and time spent on data retrieval, minimize the probability of human error, and grant immediate access to the data to authorized personnel from anywhere and at any time.

### 1.2 The used remote access technique

Global System for Mobile Communication (GSM) is currently the most commonly used communication technique because it features the best network capability and global roaming facilities. Therefore, GSM-based remote system implementations are reliable methods and are used in many applications worldwide [12].

Jose and Malekian [13] introduced a wide-ranging description of applications of GSM or mobile-based home automation systems. GSM also plays a vital role in health observation sensors and wireless communication [14]. Data transmitted from the control system of a device to the cloud or any shared space require GSM access or periodic wireless transmissions via WLAN to help establish smart cities or industries [15]. Because of the multiple uses of GSM applications, a GSM network with a Dedicated Access Point Name (DAPN) served as the primary method to transfer data from the Trimble NetR9 GNSS reference receiver.

#### 1.2.1 The concept of Dedicated Access Point Name (DAPN)

An Access Point Name (APN) is the name of a gateway and an access point onto the Internet (an IP network) from a mobile network. DAPN is a configurable network identifier used for connecting to a private GSM network. Private Mobile Connection–Dedicated APN refers to a dedicated APN with a set of features allowing the isolation of data traffic between specified private static IP addresses that are provided by the American multinational conglomerate holding company for mobile Telephone services and Telecommunications company (AT&T) [16]. DAPN enables the routing of all the data via a private VPN tunnel to the enterprise servers, and higher security is maintained via the use of a dedicated APN.

To achieve a remote connection with a GPS receiver, first, private APNs need to be configured through the gateway General Packet Radio Service support node, Packet Data Network Gateway (GGSN/P–GW), and the Domain Name System servers. Specific APNs are assigned for the end-users of the devices [17]. DAPN offers more security than public APNs, which use the Internet. Only SIM subscriptions authorized by the customer may be provisioned onto an APN gateway [18]. Other users cannot access the DAPN because it is a private network that can only be accessed by the customer's SIM subscription [19]. The DAPN solution offers truly secure mobile connectivity, comparable to the level of protection applied in private networks that allow sending customer organizations' data traffic within a closed and private group of hosts [20].

## 2 Materials and methods

**Figure 3:**
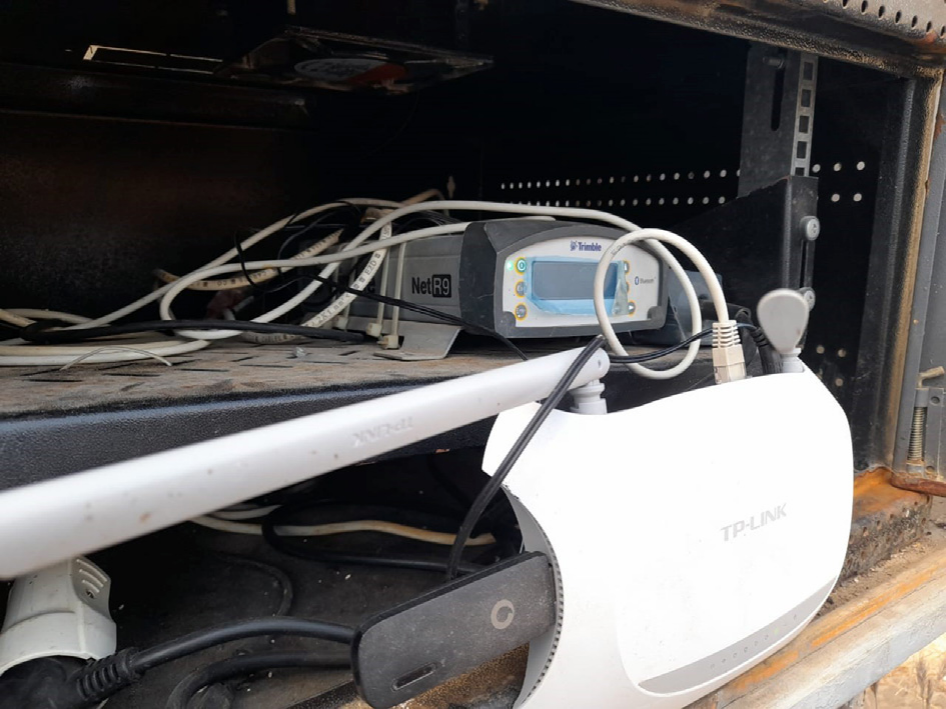
Trimble NetR9 GNSS reference receiver with remote connections.

CoRI has a multiple frequency Trimble NetR9 GNSS reference receiver installed at the Rosetta research station as shown in Figure 3. GNSS is currently the best and most innovative system available [21] and can track all GPS bands (L1/L2/L5) [22,23]. This system is installed in coastal areas to measure vertical land motions and real-time kinematic (RTK) corrections. The RTK positioning given by a single GNSS receiver only outputs an accuracy from a few centimeters up to 0.1 m near a base station [24]. Actual coordinates within a localized reference frame are delivered via high-precision GNSS positioning [25]. A tide gauge is the best method to fetch the associated climate components. Consequently, tide gauges are used to correct for the vertical land displacement component with the observed variation in the sea level [26]. The vertical land motion is a significant parameter and is thought to be impacted by climate change [27]. The GPS receiver is installed onshore near the tide gauge station to measure land displacement at the location where the gauge station measures the sea-level changes [28].

### 2.1 Configuration and set up of the system

**Figure 4:**
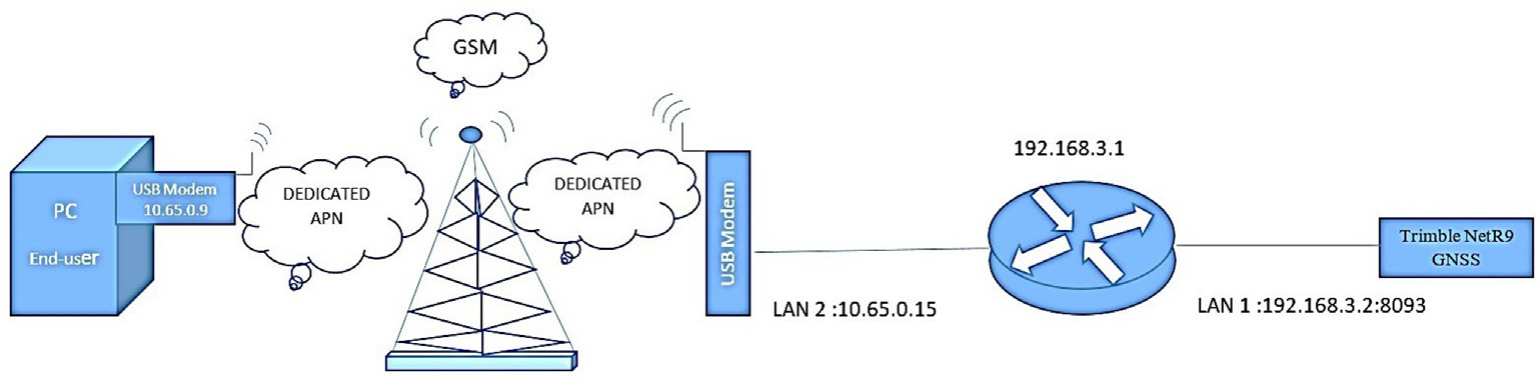
Proposed network scheme.

Setting up a public IP on the NetR9 requires remote and off-network access to the Net R9 Web User Interface (UI). An IP that can be accessed from outside the private network is called a public IP. To set up a public IP on the NetR9, the NetR9 needs to have network access and a static IP address. Figure 4 illustrates the proposed remote access scheme and solution. First, a static IP address needs to be set on the NetR9 using the device interface; the IP address is (192.168.3.2), and this IP must match the first three numbers of the network IP address.

The NetR9 Web UI can be set once the NetR9 has a public IP address. It is necessary to set a port for the Web UI to forward information to the private IP address and port. On the other side, it is necessary to set up a private IP; meanwhile, the public UI needs to be set up from the router interface. The workflow is different for every router; however, the basic principles for setting up a port-forwarding rule creates a private IP for a specific port number. Port forwarding takes the information on a particular port and forwards it to the router's private IP address. Every router is set up with a unique private IP address; however, we need to train the router to make a particular port privately available. Simultaneously, port forwarding is set up on a USB modem involving a DAPN SIM card. The static IP address is obtained from the service provider. The SIM subscription needs to be configured on the specific APN gateway and the APN settings need to be updated in the IoT device with a private IP address (10.65.0.15). Then, all the data are routed via a private VPN tunnel to the enterprise GSM servers. Finally, the Trimble NetR9 GNSS reference receiver can be smoothly accessed through the connection via the DAPN SIM card within the same private network using an IP address (10.65.0.9). This can be achieved by logging into the static private IP (10.65.0.15:8093).

## 3 Results and performance analysis

The suggested network-assisted DAPN scheme is beneficial for remote data access by authorized employers. The retrieved real-time data from the Trimble NetR9 GNSS reference receiver contains the uncorrected locations of the GPS position. The coordinates are based on the receiver's location via an autonomous and, if enabled, “submeter” approximate location. The receiver collects data on its position throughout the session based on the specified logging. Once it is done, the software uses its location and the data from the closest three Continuously Operating Reference Stations. It uses these positions to triangulate a location of the GPS receiver that has an accuracy of less than a centimeter [29]. In brief, without processing the logged data, the coordinates for the point can fall anywhere within a 3–4-foot radius of the precise location, while the processed coordinates of the point have an accuracy of less than a centimeter.

**Figure 5:**
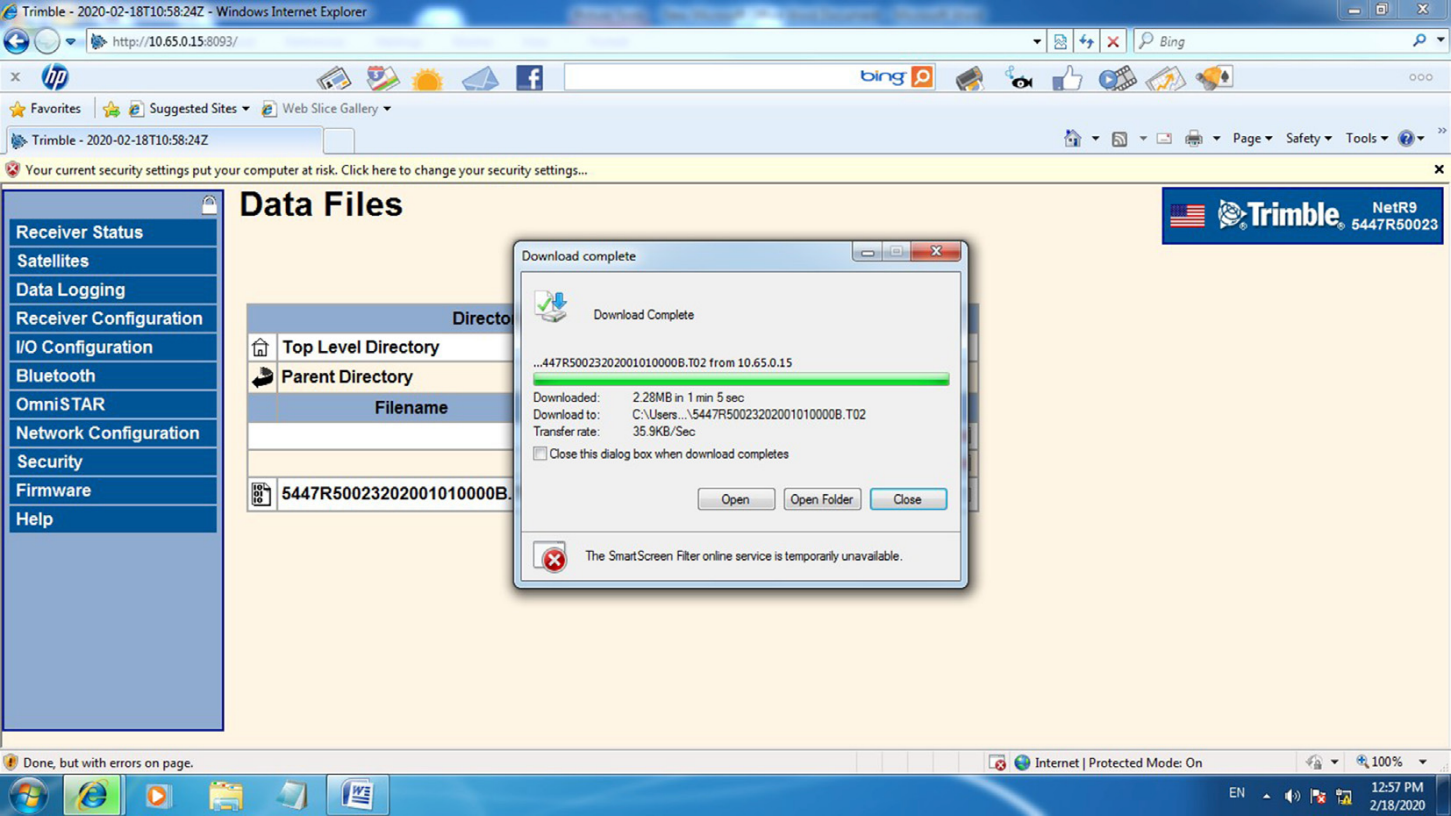
Recorded files of the Trimble NetR9 GNSS reference receiver interface download.

The coordinate time series of the station was processed using the Trimble Business Center (TBC) software to correct the required adjusted heights. Figure 5 shows the real-time remote data retrieval from the receiver with a private dedicated APN IP (10.65.0.15) and port (8093).

### 3.1 Vertical displacement

Heights or vertical displacements can be measured using numerous different methods. One method is described by a line parallel to the geoid surface at a specified point [30]. GPS receivers cannot directly provide orthometric heights. Rather, they produce heights relative to the WGS 84 ellipsoid. Therefore, the heights produced with a GPS are known as ellipsoidal (or geodetic) heights [31].

**Figure 6:**
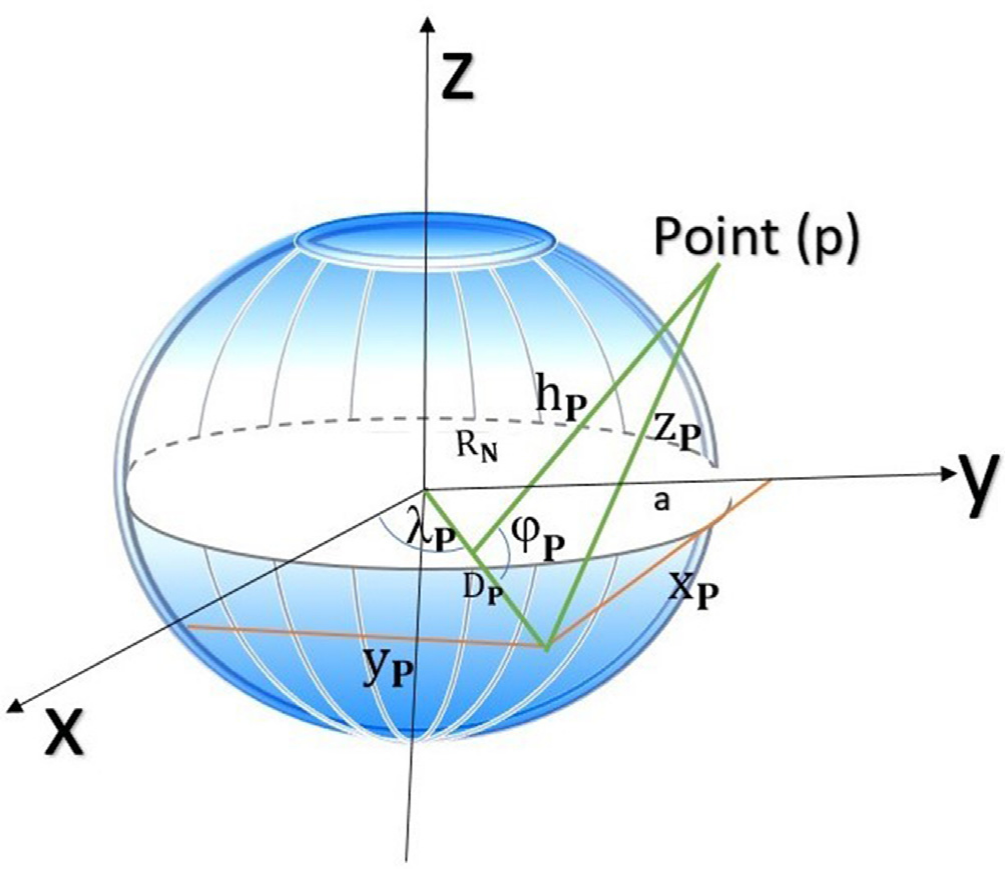
Ellipsoidal coordinate system (φ, λ, h).

From Figure 6, we can obtain

(9) $${Y_{P}}^{2}={X_{P}}^{2}+{D_{P}}^{2}-2X_{P}D_{P}\cos\lambda_{P}$$

(10) $${D_{P}}^{2}={X_{P}}^{2}+{Y_{P}}^{2}$$

(11) $$\mathrm{SO}\text{,}\;\lambda_{P}=\arccos\left[\frac{{X_{P}}^{2}+{D_{P}}^{2}-{Y_{P}}^{2}}{2X_{P}D_{P}}\right]$$

(12) $${Z_{P}}^{2}={\left(R_{N}+h_{P}\right)}^{2}+{D_{P}}^{2}-2\left(R_{N}+h_{P}\right)D_{P}\cos\varphi_{P}$$

The height (h_p_) can be calculated by substituting the appropriate values from Eqs. (6), (7), and (10) into Eq. (12).

**Figure 7:**
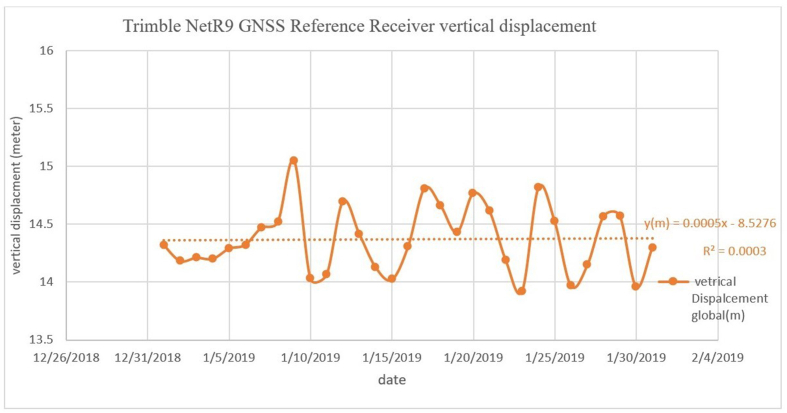
Unprocessed real-time data from the GPS station in Rosetta.

Figure (7) shows a sample of uncorrected vertical land displacement data from the Rosetta station in January 2019, where *R*^2^ (unprocessed) is equal to 0.0003.

**Figure 8:**
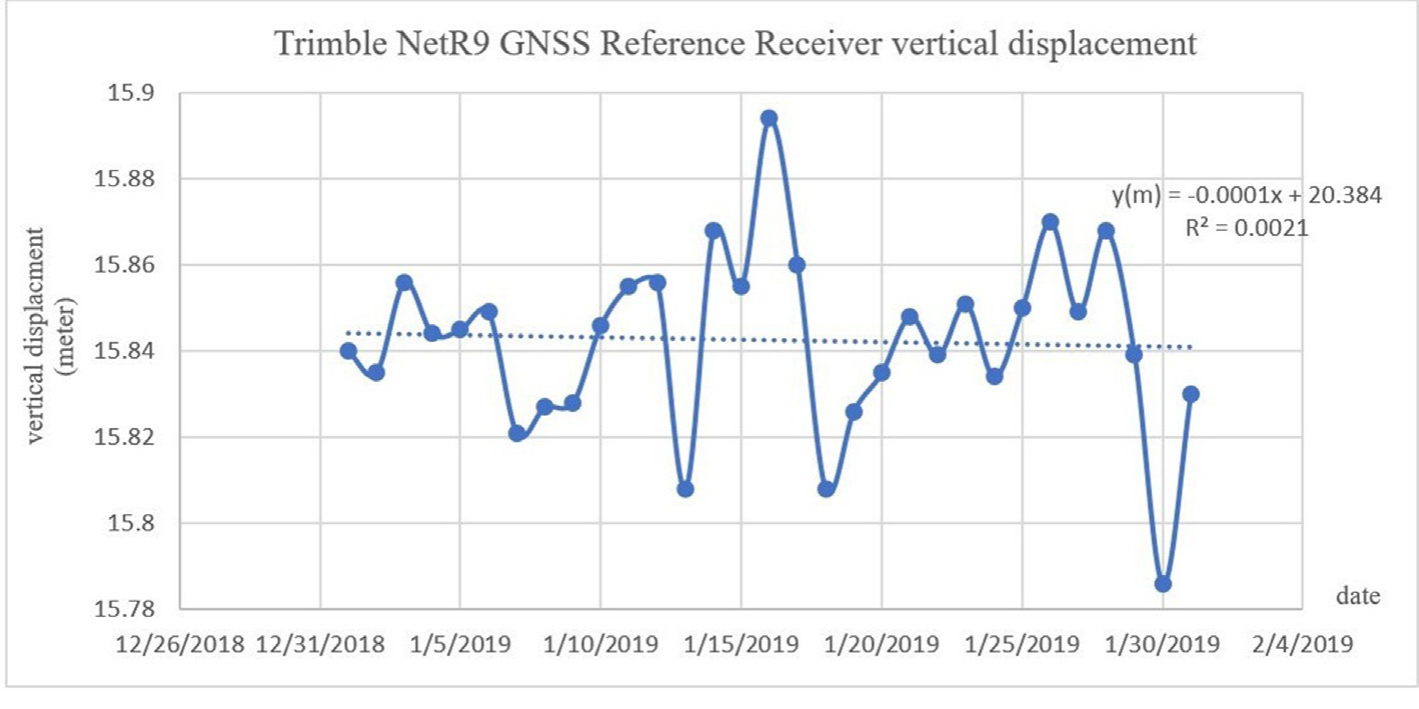
Processed real-time data for the GPS station in Rosetta.

The improved real-time data after the correction process is shown in Figure 8. The chart shows that the trend line decreases throughout January 2019 by approximately 2 mm/month. *R*^2^ here is 0.0021. The error decreased; the standard deviation of the error between the unprocessed and corrected data is 0.02, while the root mean square of the error is 0.12. These rates are strongly correlated to the rates mentioned by Becker et al., who concluded that the subsidence in the northeastern Nile Delta had a slower rate of 2–6 mm/yr [32]. In addition, Jean-Daniel et al. indicated that the Egyptian coastal vertical land displacement accounts for a variable average rate of ∼3.7 mm/yr [33].

The Statistical Product and Service Solutions (SPSS) software was used to further study the statistical results. SPSS is a bundle of software for presenting, manipulating, and analyzing data [34]. The t-test was applied in this study for the data analysis. This test offers information concerning the results of the comparisons and the significant differences between the means of two groups that may be related. The most important element of reporting the t-test results is its statistical significance [35]. There are three tables that can be used to conduct the paired samples t-test via SPSS. These involve the paired samples statistics, paired samples correlations, and paired samples tests. Each entered variable has a univariate descriptive statistic (a mean, sample size, standard deviation, and standard error) in the paired samples statistics table. The table indicates that the sample size is 31. The paired t-test can only use cases that have nonmissing reads for both variables. The paired samples correlation table shows the Pearson correlation coefficient depending on two variables (with a two-tailed test of significance) for every pair of entered variables. The paired samples test provides the postulate test results.

Table 1 shows the variation in the means of the vertical displacements before and after processing; the average is higher after processing. In addition, the standard deviation and the standard deviation error mean were improved by the processing condition.

**Table 1:** Paired samples statistics of the vertical displacements before and after processing (2019).

	Mean	N	Std Deviation	Std Error Mean
Displacement before processing	14.4	31	0.29	0.052
Pair 1				
Displacement after processing	15.8	31	0.02	0.0038

Table 2 shows the paired samples correlation statistics results. The correlation between the two variables is a single number that describes how related they are to each other. The paired samples correlation table indicates that the displacement before and after processing are significantly negatively correlated (r = −0.15). This is a weak correlation between the displacements before and after processing. This is due to the significant contrast between the displacement values before and after processing.

**Table 2:** Paired samples correlations of the vertical displacements before and after processing (2019).

	N	Correlation	Sig
Displacement before processing & Displacement after processing	31	-0.15	0.44

Table 3 illustrates the paired samples t-test results. The t statistic is evaluated by dividing the mean difference by its standard error, which is −1.4725/0.0526. The test resulted in a t-value of −27.9. A negative t-value indicates a reversal in the directionality of the effect, which has significance for the difference between two groups. This value is evidence that there is a significant difference in the data before and after processing. This value corresponds to a p-value or a two-tail significance value of 0. This is a significant result for any possible alpha level. The standard alpha level is 0.05, and resulting p-value is less than 0.05. To conclude, it is preferable to process the data after receiving it from the GPS receiver to make an appropriate analysis.

**Table 3:** Paired samples test of the vertical displacements before and after processing (2019).

	Paired differences					t	df	Sig (2-tailed)
	Mean	Std Deviation	Std Error Mean	95% confidence interval of the difference				
				low	upper			
Displacement before processing Displacement after processing	-1.4725	.293	.055	-1.58	-1.365	-27.9	30	0

## 4 Conclusions

This study introduced a new approach for the remote access of a land subsidence station using a GSM network as a means of communication to retrieve its data. A Trimble NetR9 GPS receiver station is installed at the Rosetta research station affiliated with CoRI. DAPN, through GSM, was used to promote the connection through the GSM network. DAPN has Transparent End-To-End Connectivity, with a secure mobile virtual private network, and VPN (IPSEC) designed for global cellular IoT deployments. This enabled instantaneous remote access of the recorded data instead of site visits. This helps decrease costs and time wasted obtaining data, reduces the probability of human error, and ensures immediate access from anywhere. The NetR9 GPS receiver station collects the data in an unprocessed (uncorrected) form. This is because the receiver collects data on its position throughout the session without referencing the three-dimensional coordinates.

The results of the study demonstrate the apparent difference between the GPS receiver retrieved real-time displacement data before processing (global point) and the data after processing throughout January 2019. The processing was done using the TBC software. The *R*^2^ value was improved after the correction: it was 0.0003 before the correction and 0.0021 after the correction. The root mean square of the error between the pre- and postprocessing of the data is 0.12, and the standard deviation of the error is 0.02.

SPSS was used to emphasize the analysis of the displacement results. Paired samples t-tests are commonly used to test the statistical difference between two measurements. The paired samples statistics provide univariate descriptive statistics (mean, sample size, standard deviation, and standard error) for each variable entered. The paired samples test was conducted to show that the average of the vertical displacement was higher after the correction process. In addition, the standard deviation and the standard deviation error mean were enhanced after the correction. The t-value indicated that there was a significant difference between the data before and after the correction process. Therefore, it is recommended for researchers to process the data before making analyses.

The results of this study can be applied at a global scale because most of the world's deltas are suffering from alterations caused by different factors such as sea-level rise, land subsidence, land inundation, and sediment flux deficiency. Decreases in the sediment load in addition to land subsidence adversely affect river deltas because they induce shoreline retreat and land loss. The Colorado River Delta in Mexico, Indus River Delta in Pakistan, Ibro River Delta in Spain, Krishna River Delta in India, and Yellow River Delta in China have all shown sediment decreases of approximately 90%.

## Declarations

### Author contribution statement

Salsabeel E. Othman: Conceived and designed the experiments; Performed the experiments; Analyzed and interpreted the data; Contributed reagents, materials, analysis tools or data; Wrote the paper.

Hesham. F. A. Hamed: Analyzed and interpreted the data; Contributed reagents, materials, analysis tools or data.

Gerges M. Salama: Conceived and designed the experiments; Analyzed and interpreted the data; Contributed reagents, materials, analysis tools or data.

### Funding statement

This research did not receive any specific grant from funding agencies in the public, commercial, or not-for-profit sectors.

### Data availability statement

The authors do not have permission to share data.

### Declaration of interests statement

The authors declare no conflict of interest.

### Additional information

No additional information is available for this paper.
